# Origin-Independent Densities of Static and Dynamic Molecular Polarizabilities

**DOI:** 10.1021/acs.jpclett.1c02545

**Published:** 2021-09-08

**Authors:** Francesco Ferdinando Summa, Guglielmo Monaco, Paolo Lazzeretti, Riccardo Zanasi

**Affiliations:** † Dipartimento di Chimica e Biologia “A. Zambelli”, Università degli studi di Salerno, via Giovanni Paolo II 132, Fisciano 84084, SA, Italy

## Abstract

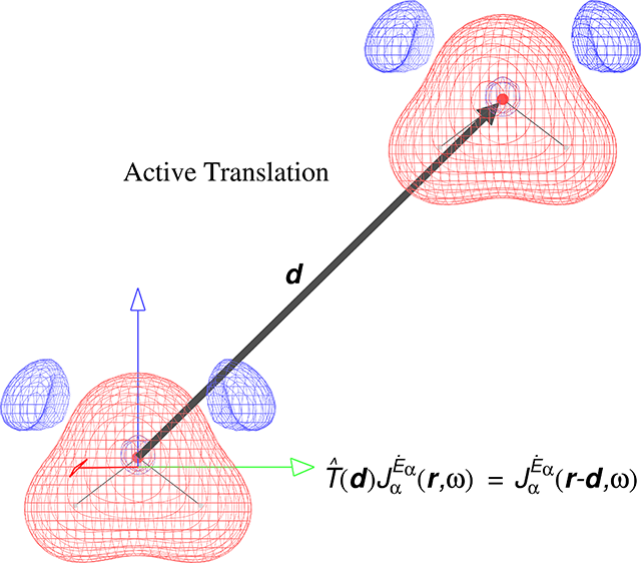

The notion of the electric dipole polarizability density function of atoms and molecules has been considered. The current density induced by the time derivative of the electric field of monochromatic light allows for a new definition of the electric dipole polarizability density, which is translationally invariant. This translational invariance provides the physical meaning that was lacking in previous defined polarizability densities. The new polarizability density has been implemented at the TD-DFT level of theory. The origin independence has been proven *in silico* to hold regardless of the basis set size. Two emblematic molecules, i.e., CO and N_2_, which in many respects display similar electric response, have been studied in detail. The substantial differences, which have been highlighted in the topology of the parallel and perpendicular polarizability density tensor components of CO and N_2_, are grossly hidden by compensation, when integration is carried out to get the molecular properties.

A beam of light induces oscillating charge and current distributions in the electron cloud of a molecule.^1,2^ Assuming for the sake of simplicity that the impinging radiation is a monochromatic plane wave of frequency ω, these periodic oscillations can be expressed in terms of dynamic electric polarizabilities and hyperpolarizabilities, i.e., response tensors of increasing rank, explicitly depending on ω. Within the long-wavelength approximation,^3,4^ the leading contribution to light scattering is provided by the oscillating electric dipole ***μ***(*t*) induced by the electric field of the monochromatic wave, whose strength ***E***(*t*) = ***E***_0_ cos(*ωt*) is assumed to be spatially uniform all over the molecular domain. If we limit ourselves to consider linear response, the induced dipole is expressed by the relationship
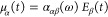
where the second-rank polar tensor α_*αβ*_(ω), symmetric under the exchange α ↔ β, represents the frequency-dependent electric dipole polarizability.

Static, i.e., α_*αβ*_(0) ≡ α_*αβ*_, and dynamic α_*αβ*_(ω) are possibly the most intensively studied linear-response properties of atoms and molecules, at the experimental as well as the theoretical level. They are used to rationalize basic optical properties, e.g., refraction indices and absorption coefficients.^5−9^ Moreover, the Rayleigh scattering depolarization ratio of gases depends on molecular polarizability anisotropy.^10^

The Stark effect is observed in the pure rotational spectrum of molecules and in the electronic spectra of atoms.^11^ Static polarizability anisotropies of linear and symmetric top molecules, determined by Alms and co-workers measuring the depolarization ratio of Rayleigh scattered light,^12^ can also be determined from the second-order Stark shift in microwave spectroscopy.^13^

Within the theory of intermolecular forces and long-range interactions,^2,14,15^ electric polarizabilities are related by perturbation theory to the charge distribution and polarizabilities of the isolated monomers.^16^

A much investigated problem, yet unsolved to a satisfactory extent in the theory of electric polarization, is that of determining the main contributions to the induced dipole ***μ***(*t*) in the presence of ***E***(*t*) in relation to diverse regions of a molecule. In order to understand mechanisms and causes that come into play to bring about polarization of the electron cloud and to understand the role of different substituents and moieties entering a given molecular substrate, and related push–pull effects, attempts have been made to define reliable resolutions of the electric dipole polarizability into contributions arising from various molecular domains, with the aim that they be movable from molecule to molecule. Accordingly, partitions into transferable additive terms have been defined allowing for various criteria.^17−22^ A simple scheme for separating molecular polarizability into atomic and bond contributions has been obtained by employing the electric dipole acceleration gauge.^17,19^

A valid and rigorous approach has been proposed relying on powerful tools provided by the quantum theory of many-particle systems,^23^ introducing a dynamic charge density susceptibility which has been decomposed using regular solid harmonics.^20^

The related notion of nonlocal polarizability density, first introduced by Maaskant and Oosterhoff,^24^ has been widely discussed and applied by Hunt and co-workers in a series of papers.^25−30^ Sipe and Van Kranendonk analyzed some limitations of the concept of polarizability density applied to atoms and molecules.^31^ Polarizability densities within simple one-electron atomic systems have been investigated by Orttung and Vosooghi.^32^

The general concept of a density function for a molecular electronic property has been considered by Jameson and Buckingham, who investigated the origin dependence of some electric and magnetic property densities.^33^ The nuclear magnetic shielding density function was used as a typical example.^34^

Frequency-dependent distributed polarizabilities have been computed by time-dependent Hartree–Fock (TDHF) calculations by Hättig et al.,^20^ within Bader’s theory of atoms in molecules (AIM).^35−37^ Recently, an origin-independent decomposition of static polarizability based on AIM has been put forward by Montilla and co-workers.^21^ Alparone reported calculated polarizabilities and hyperpolarizabilities obtained integrating origin-dependent perturbed electron densities, at first and second order, respectively, employing a Coulomb-attenuating Density Functional Theory (DFT) method.^38^

A Hirshfeld-based scheme for resolving the intrinsic polarizability densities has been proposed by Otero and co-workers,^39^ and a partition into fluctuating charge and induced atomic dipole contributions has been put forward by Mei et al.^40^ An extension to consider also hyperpolarizability density has been advanced and applied by Nakano et al.,^41^ Yamada et al.,^42^ and Alparone.^38^

Taking into account this state of affairs, we are concerned in this article with a novel theoretical method for computing and representing polarizability density functions of position ***r*** in molecules, via planar maps and 3D-perspective views of isosurfaces, quite useful to indicate molecular regions most involved in the electric field-induced polarization of the electron cloud.

Whereas approaches presented so far^38,39,41,42^ yield visualizations of polarizability density depending on the origin of coordinate system, which reduces their physical relevance, the computational technique outlined in this work provides an effective, translationally invariant, definition of static and dynamic polarizability densities.

Here in the following we outline the notation and the theoretical method. Within the Born–Oppenheimer (BO) approximation,^43^ for a molecule with *n* electrons and *N* clamped nuclei, charge, mass, position, and canonical momentum of the *k*-th electron are indicated, in the configuration space, by −*e*, *m*_e_, ***r***_*k*_, ***p̂***_*k*_, *k* = 1, 2, ..., *n*, using boldface letters for electronic operators. Analogous quantities for nucleus *I* are *Z*_*I*_*e*, *M*_*I*_, ***R***_*I*_ for *I* = 1, 2, ..., *N*.

The imaginary unit is represented by a Roman i. Throughout this paper, SI units are used and the standard tensor formalism is employed; e.g., the Einstein convention of implicit summation over two repeated Greek indices is applied. The third-rank Levi–Civita pseudotensor is indicated by ϵ_*αβγ*_.

Capitals denote *n*-electron vector operators, e.g., for position and canonical momentum,
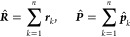
Then the electric dipole operator in the length formalism becomes ***μ̂*** = −*e****R̂***.

Expressions for the polarization charge density and current density induced in the electrons of a molecule by a monochromatic plane wave are obtained by time-dependent quantum mechanical perturbation theory,^44^ assuming that the eigenvalue problem for the time-independent BO electronic Hamiltonian *Ĥ*^(0)^Ψ_*j*_^(0)^ = *E*_*j*_^(0)^Ψ_*j*_^(0)^ has been solved, determining a set of eigenfunctions Ψ_*j*_^(0)^ and corresponding energy eigenvalues *E*_*j*_^(0)^. The reference (ground) state is indicated by Ψ_*a*_^(0)^ and the natural transition frequencies are ω_*ja*_ = (*E*_*j*_^(0)^ – *E*_*a*_^(0)^)/*ℏ*.

Let us introduce the general definition of *n*-electron density functions^45^
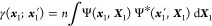
1of electronic space-spin coordinates ***x***_*k*_ = ***r***_*k*_ ⊗ ***s***_*k*_, *k* = 1, 2, ..., *n*, where
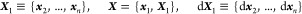
2Thus, integrating over d*s*_1_, one gets from eqs 1
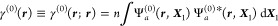
3for the reference (ground) state Ψ_*a*_^(0)^ of the molecule. The probability current density^45^ is obtained from eqs 1–3 for the density matrix,
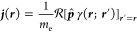
4In this relation, one puts ***r***′ = ***r*** after operating with the electronic canonical momentum ***p*^** = −iℏ∇. The interaction Hamiltonian considered in the present work does not contain terms depending on electron spin; therefore the probability current density (4) includes only orbital contributions. The corresponding charge current density is obtained multiplying by −*e*, i.e., ***J*** = −*e****j***.

Let ρ^(0)^(***r***) = −*eγ*^(0)^(***r***) be the electronic charge density in the absence of perturbation. The time-dependent electric field ***E***(*t*) carried by a monochromatic plane wave with frequency ω, assumed spatially uniform within the electric dipole approximation,^3,4^ induces an oscillating polarization of the electronic distribution. If the intensity of ***E*** is weak enough, first-order time-dependent perturbation theory^44^ can be applied to describe the interacting system.^46^ For instance, the total electronic charge density can be expressed as a truncated series
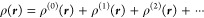
5introducing polarization densities of increasing order induced by the perturbation, for example,
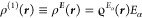
6where
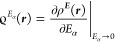
7is described by a vector function of position for any value of ω, including 0 for a static electric field,^47^
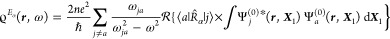
8To first-order in ***E***, the electric dipole moment induced in the electron distribution is given by
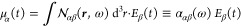
9where  is a polarizability density tensor component defined as
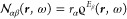
10

In eq 9 we have identified the integral with the second-rank tensor
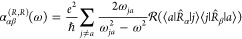
11symmetric in the α and β indices, i.e., with the frequency-dependent electric dipole polarizability in the dipole length gauge.

The product of vectors
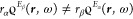
12in the integral (9) for the induced moment defines a *polarizability density tensor function*(47) of position ***r***, which is not symmetric in the α and β indices. These definitions of electric polarizability density and electric dipole moment density were used by Theimer and Paul to calculate anisotropic light scattering in a dense monatomic gas^48^ and have been discussed by Jameson and Buckingham.^33^

It is easily understood that the space integral of the polarization density vector (8) all over the molecular domain vanishes, due to orthogonality of the eigenstates Ψ_*a*_^(0)^ and Ψ_*j*_^(0)^, thus fulfilling the constraint of charge conservation,^47^ i.e.,
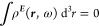
13Therefore, the integral (9) for μ_α_(*t*) is independent of the origin of the *r*_α_ vector. Accordingly, no such origin appears in this equation.

However, the components *r*_α_ and *r*_β_ of the position vector depend on the origin ***r***′ chosen for the coordinate system, and change in a passive parallel translation represented by the arbitrary shift ***d***,
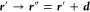
14Therefore, the polarizability density (10) also varies in plots^38,39,41,42^ obtained using different coordinate systems. For this reason, visualizations reported so far for the polarizability density tensor based on eqs 8, 9, and 10 are of doubtful physical meaning and computationally impractical.

A more promising approach is available within the framework of recent proposals, introducing the interaction Lagrangian density and a perturbative expansion for the moments of the polarization charge density function, in connection with corresponding moments of the current density, via the general relationship^46,49^
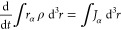
15

In the present context, we will make use of eq 15, allowing for the definition of electronic charge density vector (6)–(8) and current density vector **J^*Ė*^** induced by the time derivative ***Ė*** of an optical electric field.^46,47^ Thus, more viable computational procedures are based on a dynamic, second-rank current density tensor (CDT), obtained by differentiation of the current density vector,
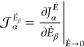
16expressed in the form^46^
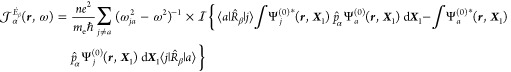
17

Actually, in agreement with eq 15, the translationally invariant CDT defined via eq 17 can be interpreted as a *polarizability density function*,^47^ alternative to—and more meaningful from the physical viewpoint than—the widely adopted (12), since
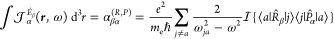
18where α_*βα*_^(*R*,*P*)^ is the electric dipole polarizability in mixed dipole length-dipole velocity formalism. It is identical with (11), if the off-diagonal hypervirial relationships^50,51^
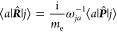
19are satisfied.

Incidentally, it is worth recalling that off-diagonal α_*βα*_^(*R*,*P*)^ tensor components are symmetric in the exchange α → β only if the hypervirial theorem (19)^50,51^ is fulfilled, i.e., in the case of exact eigenfunctions to a model Hamiltonian and optimal variational eigenfunctions.^52^ Within the algebraic approximation,^53^ in the absence of molecular point group symmetry, the identity α_*βα*_^(*R*,*P*)^ = α_*αβ*_^(*R*,*P*)^ is satisfied only in the limit of a complete basis set. The degree to which it is fulfilled, together with α_*αβ*_^(*R*,*P*)^ = α_*αβ*_^(*R*,*R*)^, provides simple tests for basis set quality.^54^

Using one more time the off-diagonal hypervirial relation, i.e., substituting (19) in (17), one obtains
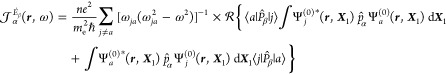
20which integrates to
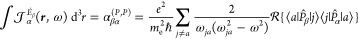
21where α_*βα*_^(*P*,*P*)^ is the symmetric electric dipole polarizability tensor in the velocity formalism.

The great advantage offered by definitions (17) and (20) with respect to (10) is immediately evident, in that they are *invariant of the origin*. They are valid for any value of ω, including ω → 0, i.e., for a static electric field. Plots of densities (17) and (20) are expected to provide fundamental information on the polarization of the electron cloud.

The tensor  is connected with two physical quantities, depending on whether it is multiplied by ***E***(ω, *t*) = ***E***_0_ cos(*ωt*) or 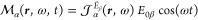
22
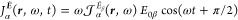
23

These relations define the dipole density vector  induced in the molecule and the current density vector *J*^*Ė*^(***r***, ω, t), quite important for the interpretation of static and dynamic properties. In the static case we have ***E***(0, *t*) = ***E***_0_ and ***Ė***(0, *t*) = 0; therefore, visualizations of the polarizability density provide important information on the dipole moment induced in the molecule. For ω ≠ 0, eq 23 defines the current density vector induced by an electric field out of phase of π/2.

The theoretical formulation of the polarizability density function described above can be straightforwardly implemented within the random phase approximation (RPA) formulation of the TD-HF^55−57^ and TD-DFT^58−61^ frameworks. For that purpose, we rewrite eqs 10, 17, and 20 in the forms
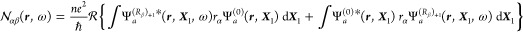
24
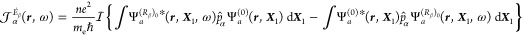
25
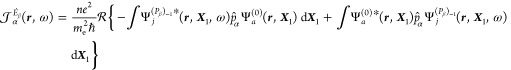
26where
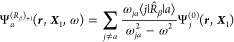
27
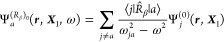
28
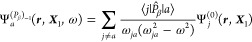
29can now be regarded as perturbed wave functions explicitly dependent on the radiation frequency. For a more general definition of dynamic perturbed wave functions and meaning of the various symbols, see ref (62).

For a closed-shell system, in the one-determinant approximation assuming real molecular orbitals, eqs 24, 25, and 26 take the forms that have been coded in atomic units:
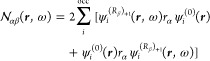
30
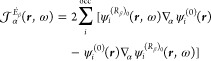
31
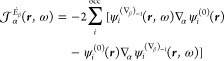
32In the equations above and in the following, the indices *i* and *m* are used for occupied and virtual orbitals, respectively, and *q* denotes basis set functions. Then, orbitals ψ_*i*_ are expanded as linear combinations of basis set functions χ_*q*_
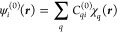
33
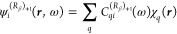
34
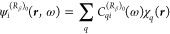
35
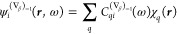
36where *C*_*qi*_^(0)^, , , and  are molecular orbital expansion coefficients. The superscript (0) indicates canonical unperturbed coefficients, whereas
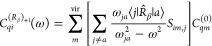
37
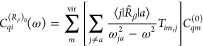
38
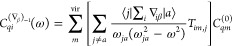
39are frequency-dependent perturbed coefficients. In our implementation, transition amplitudes ***T***_*j*_ and corresponding transition energies ω_*ja*_ are obtained by means of a TD-DFT (or TD-HF ≡ RPA) calculation. In particular, the ***T*** matrix is defined as in ref (57) and is determined from the standard amplitudes ***X*** and ***Y***(59−61) as . The utilities contained in the Gaussian interfacing package have been used to obtain ***X*** and ***Y*** in machine precision. The full procedure, for the calculation of the frequency-dependent electric dipole polarizability density, has been implemented within the freely available SYSMOIC^63^ program package.

In order to show some preliminary applications of the theory exposed in this paper, three simple model systems have been considered for the calculation of static and dynamic electric dipole polarizability densities at the TD-DFT level of approximation. They are H_2_O and the linear molecules CO and N_2_. Owing to their small size, very accurate computations have been carried out, using Hartree–Fock (HF) method and the BHandHLYP functional,^64^ recently shown to provide good linear response properties,^54^ adopting basis sets of contracted functions which include terms of high angular momentum, taken from BSE.^65^ For C, N, and O atoms we have adopted the aug-cc-pV7Z, which corresponds to a (9s8p7d6f5g4h3i2j) basis set. BHandHLYP transition matrix elements of operators ***R̂*** and **∇**, amplitudes ***S***_*j*_ and ***T***_*j*_, and energies ω_*ja*_, appearing in eqs 37, 38, and 39, have been computed by the Gaussian 16 program package,^66^ using the TD = (full,sos) 6d 10f keywords. Molecular geometries were optimized at the BHandHLYP/aug-cc-pVTZ level. Polarizability densities have been evaluated using the SYSMOIC program package.^63^ Spatial integration of the density functions has been performed using the Becke scheme,^67^ adopting 131 angular points for the Lebedev’s quadrature of 59th order of accuracy^68^ and 131 radial points for the Gauss–Chebyshev radial quadrature of second kind.^69^

Let us first consider the origin independence of the polarizability densities defined by means of , see eqs 17 and 20 for the (*P*,*R*) and (*P*,*P*) formalisms, in comparison with the conventional origin-dependent polarizability density (***r***, ω), and see eq 10 for the (*R*,*R*) formalism. This is a quite interesting point since translational invariance is a fundamental requirement for any physically meaningful density, *irrespective of basis set choice*. To investigate this aspect, we have calculated the isotropic value of the polarizability densities of the H_2_O molecule for two different origins, adopting the rather small 6-31G(d,p) basis set at the TD-HF level of theory. The origin hereafter denoted “000” has been chosen by making it coincide with the center of positive charges. The second origin, referred to as “123”, has been set shifting the previous one by 1, 2, and 3 bohr along *x*, *y*, and *z*, respectively.

The results are shown in Figure 1, where three densities have been plotted as iso-surfaces of red/positive or blue/negative sign, side by side for the two origins: “000” on the left, “123” on the right. As can be observed, the first density, with label **a**, evaluated via eq 10, shows a marked origin dependence. The last two, corresponding to the (*P*,*R*) and (*P*,*P*) formalisms, are clearly origin-independent with respect to both passive and active translations. These findings are very appealing, also in consideration of the fact that translational invariance of **b** and **c** is independent of basis set quality.

**Figure 1:**
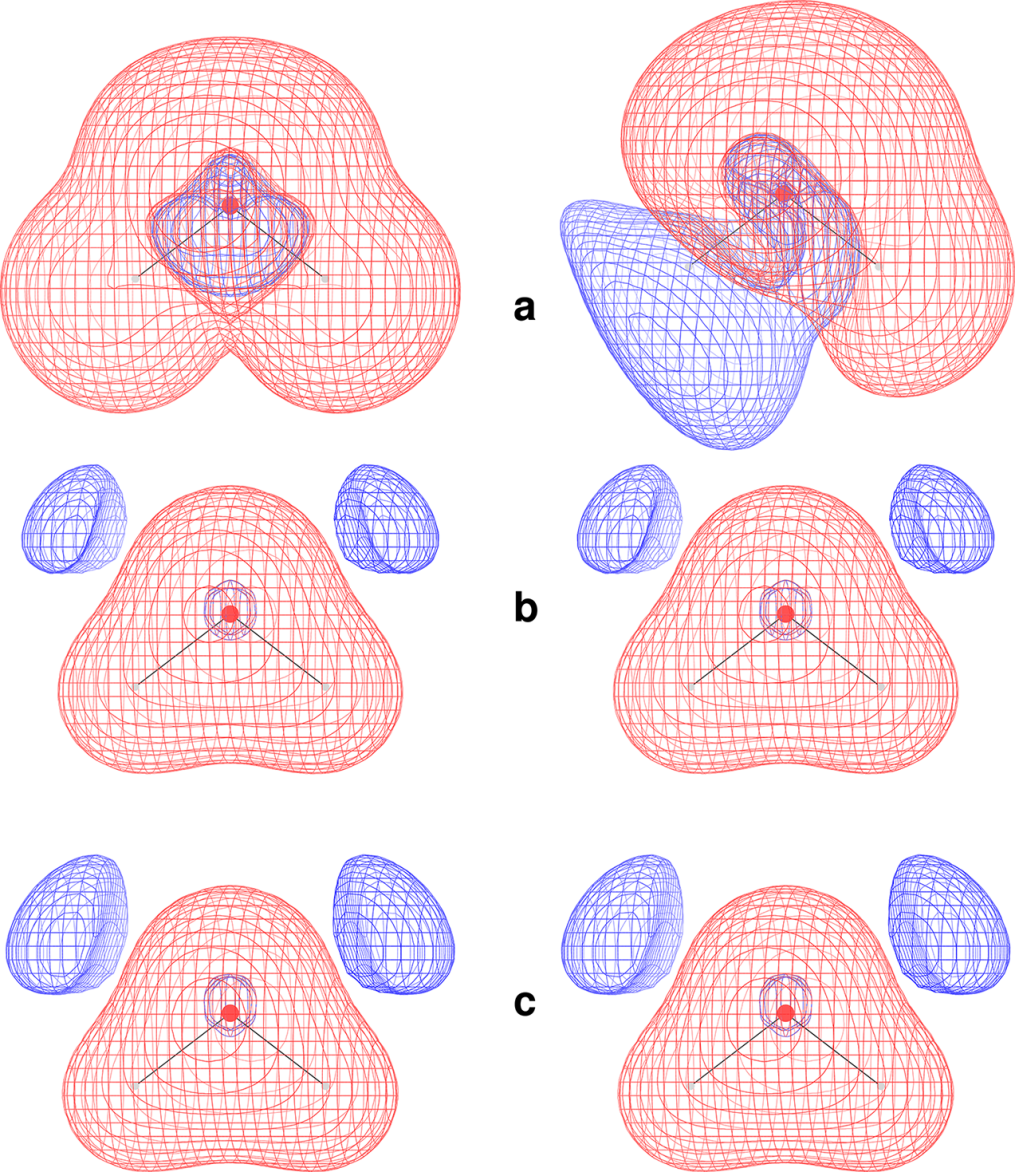
Calculated isotropic polarizability densities for the H_2_O molecule at the TDHF 6-31G(d,p) level of approximation, for a radiation frequency of ω = 0.077317 au, displayed as isovalue surfaces: red +0.02, blue −5 × 10^–4^*a*_0_^3^. Densities on the left/right column are relative to the “000”/“123” origin, see text. Labels **a**, **b**, **c** denote the three different polarizability densities defined by eqs 10, 17, and 20, respectively.

Some more points of interest are

1. Densities **b** and **c** would be exactly the same in the limit of complete basis set. In Figure 1 some differences can be observed owing to the small size of the basis set adopted.
2. Computed tensor components α_*αβ*_ depend on basis set quality. Upon integration, all densities, for both origins, would converge to the same value of dipole polarizability in the complete basis set limit.
3. The density **a** depends on the origin, although the corresponding electric dipole polarizability does not, but improves on increasing basis set quality toward the complete basis set result.

Carbon monoxide is similar to the nitrogen molecule, in that it has the same number of electrons, chemical bonds, and lone pairs. To some extent, also their electric response properties, permanent electric dipole moment, and electric dipole polarizability, are similar; consider, for instance, the very small dipole moment of CO, 0.122 D.^70^ However, these molecules contain distinct atomic species and different polarizability densities are expected to characterize them, in connection with diverse topology of induced current density fields. To highlight this point, in Figure 2 and Figure 3 we display diverging color maps for the polarizability density tensor components of CO and N_2_, respectively, calculated for the static case and for two radiation frequencies which bracket, one less and one greater than, the excitation energy associated with the first nonzero transition moment of the corresponding tensor component.

**Figure 2:**
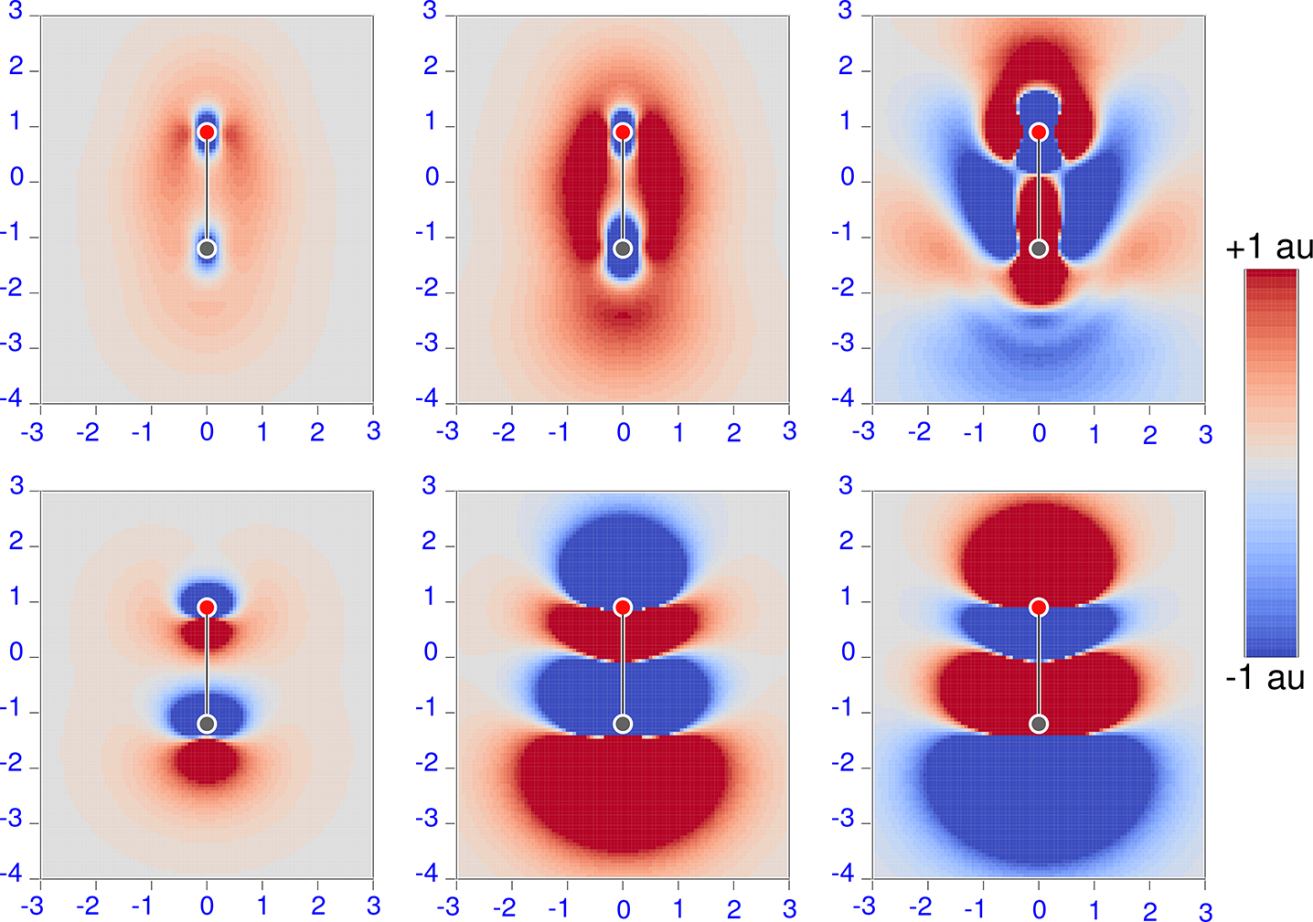
Diverging color map of the origin-independent polarizability density functions for the CO molecule. The CPK (Corey–Pauling–Koltun) color scheme colors “atom” objects by the atom (element) type.^72^ The top row shows parallel components calculated by eq 31 for three radiation frequencies, ω = 0.0, 0.393, and 0.401 au, from left to right. The polarizability components obtained by spatial integration are α_∥_^(*R*,*P*)^(0) = 14.67, α_∥_^(*R*,*P*)^(0.393) = 44.03, and α_∥_^(*R*,*P*)^(0.401) = −18.37 au. The bottom row shows perpendicular components calculated for three radiation frequencies, ω = 0.0, 0.312, and 0.325 au, from left to right. The polarizability components obtained by spatial integration are α_⊥_^(*R*,*P*)^(0) = 11.59, α_⊥_^(*R*,*P*)^(0.312) = 64.02, and α_⊥_^(*R*,*P*)^(0.325) = −73.71 au

**Figure 3:**
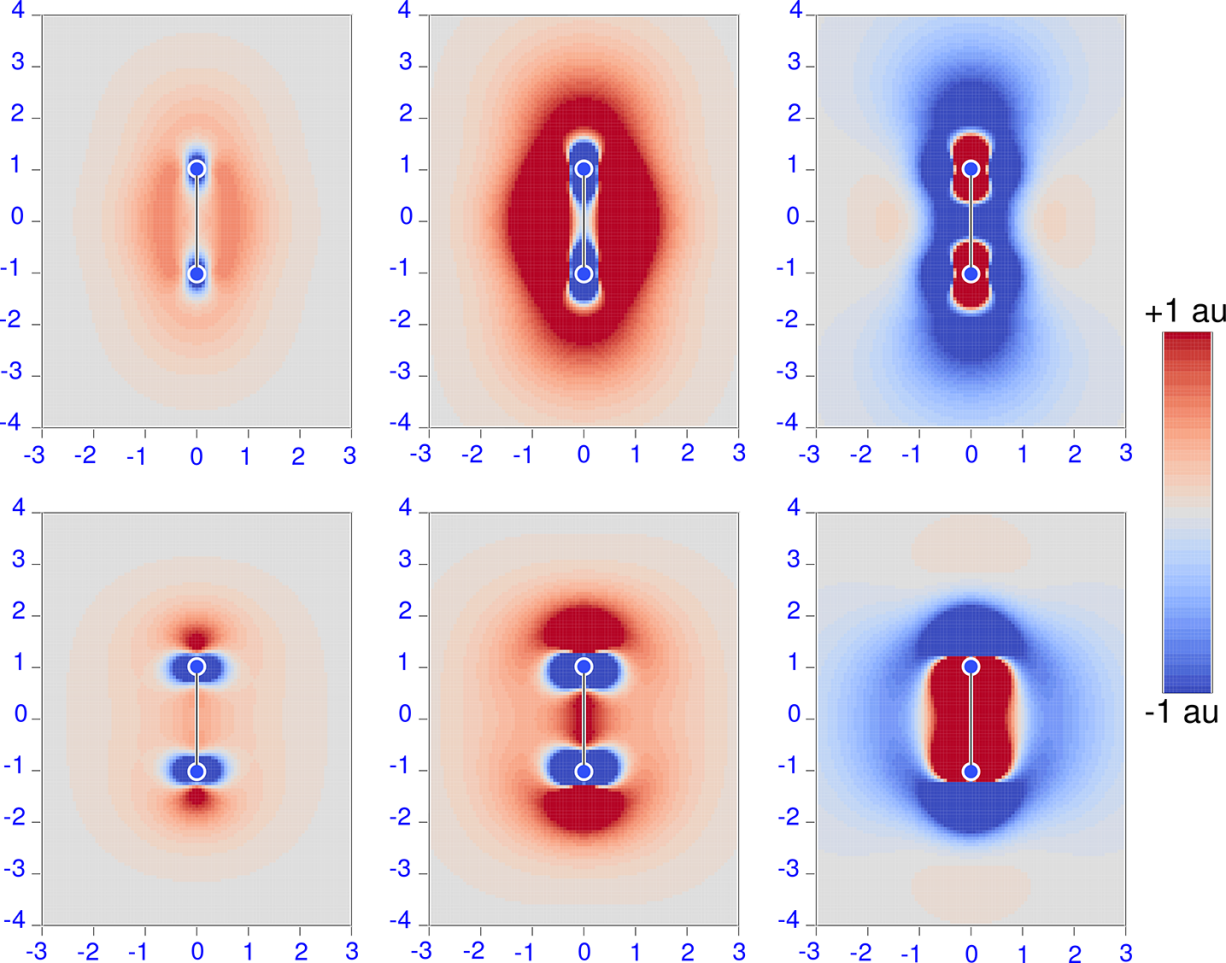
Diverging color map of the origin-independent polarizability density functions for the N_2_ molecule. The top row displays parallel components calculated by eq 31 for three radiation frequencies, ω = 0.0, 0.465, and 0.486 au, from left to right. The polarizability components obtained by spatial integration are α_∥_^(*R*,*P*)^(0) = 14.56, α_∥_^(*R*,*P*)^(0.465) = 71.89, and α_∥_^(*R*,*P*)^(0.486) = −40.92 au. The bottom row displays perpendicular components computed for three radiation frequencies, ω = 0.0, 0.450, and 0.478 au, from left to right. The polarizability components obtained by spatial integration are α_⊥_^(*R*,*P*)^(0) = 9.93, α_⊥_^(*R*,*P*)^(0.450) = 29.20, and α_⊥_^(*R*,*P*)^(0.478) = −16.40 au

For ω ≠ 0, we stress that our approach is not valid in the near-resonant region, since the sum-over-states (SOS) relations used here, eqs 11, 18, and 21, do not contain any (phenomenological) damping term representing the finite lifetime of the excited states.^71^ Therefore, we have carefully chosen the frequency values to keep the polarizability tensor components below reasonable limits within the transparent region. The captions to Figure 2 and Figure 3 report α_∥_ and α_⊥_ obtained by integrating the polarizability densities displayed. As can be observed, the absolute value of the integrated polarizability components is always less than 75 au.

For the static case, we found that the parallel polarizability densities of CO and N_2_ are characterized by similar negative domains of small extension in the vicinity of the nuclei, embedded within a positive region, which in carbon monoxide is more conspicuous about the oxygen nucleus (in the upper end of the CO bond in Figure 2) than carbon’s, while in N_2_ the obviously symmetric positive distribution has a magnitude roughly intermediate between that of O and C in Figure 2. Interestingly, the α_∥_ components calculated in this study are almost the same for CO (14.67 au) and N_2_ (14.56 au), despite the different density distributions.

The perpendicular component of the polarizability density presents pairs of “bubbles” of opposite sign near each nucleus, resembling a p orbital oriented along the bond. Notably, the bubble size depends on the nuclear species, being larger about C rather than O in carbon monoxide. In the nitrogen molecule, the bubbles have smaller size and are characterized by N-centered negative cores. In any case, positive (red) regions extend in space much more than negative (blue) ones. Moreover, the bubble pairs are aligned in CO, whereas they are antialigned in N_2_.

Remarkably, the domains of juxtaposed bubbles with opposite sign tend to offset one another upon integration. Therefore, their contribution to the integrated property almost vanishes. As a consequence, α_⊥_(0) turns out to be smaller than α_∥_(0), a result that would be difficult to explain without the corresponding maps of polarizability density components shown in Figures 2 and 3. Actually, our calculated values are 11.59 and 9.93 au for CO and N_2_, respectively, whereas computed α_∥_(0) for these molecules are 14.67 and 14.56 au, respectively.

Comparing our results with the coupled-cluster (CCSDT) static polarizability calculated by Hammond et al.,^73^ we note a general good agreement, with the relevant exception of α_∥_ in CO for which a discrepancy as large as 6% is observed. From this point of view, it seems clear that for this component the electronic correlation provides a sensible contribution, larger in CO than in N_2_, as evidenced also by the quite small dipole moment estimated here 4 × 10^–4^ D, at least with the correct sign.

For the dynamic case, the positive regions of electric dipole polarizability density are observed to increase steadily more than the negative ones, as the radiation frequency increases from zero toward the first transition energy in both molecules; see, for example, the central insets of Figure 2 and Figure 3. This is consistent with the expected accretion of the polarizability tensor components. Since the first transition energy for the perpendicular matrix element ⟨*j*|*R̂*_⊥_|*a*⟩ is less than the first transition energy for the parallel matrix element ⟨*j*|*R̂*_∥_|*a*⟩ in both molecules, α_⊥_(ω) becomes larger than α_∥_(ω) as the radiation frequency ω approaches the first transition pole, as also noted in ref (73).

The situation changes drastically when the components turn negative whenever ω goes beyond a resonant frequency. Interestingly, with the exception of the perpendicular polarizability density in CO, the density functions do not just change their sign, but undergo also a significant modification of size, as well as distortion of their shape.

Since it is conventionally accepted that velocity gauges in general show slower basis set convergence than length gauges, we have performed a brief preliminary study on the water molecule, calculating the static polarizability density tensor components using a number of basis sets of increasing quality from the same source.^74^ Results for the integrated polarizabilities are shown in Table 1, in comparison with a well-known literature data, adopting the same experimental geometry.^75^ As can be observed, the aug-pcSseg-1 basis set already provides fairly good results in comparison with those obtained with the larger basis sets. Consistently, the aug-pcSseg-1 polarizability densities for the (*P*,*R*) and (*P*,*P*) formalisms (not shown here for the sake of space) do not display any significant differences with respect to those calculated at higher levels. A more complete study on the basis set convergence for the different gauges using a few suitable chosen molecules is under way.

**Table 1:** Static HF Polarizability Components of Water for a Number of Basis Sets of Increasing Quality from the Same aug-pcSseg Family^74^

basis set	formalisms	α_*xx*_	α_*yy*_	α_*zz*_
aug-pcSseg-0	(*R*,*R*)	3.8	6.9	5.1
	(*P*,*R*)	3.5	6.3	4.7
	(*P*,*P*)	3.3	5.8	4.4
aug-pcSseg-1	(*R*,*R*)	7.8	9.1	8.4
	(*P*,*R*)	7.6	9.0	8.3
	(*P*,*P*)	7.5	8.9	8.2
aug-pcSseg-2	(*R*,*R*)	7.87	9.16	8.47
	(*P*,*R*)	7.83	9.13	8.46
	(*P*,*P*)	7.80	9.12	8.45
aug-pcSseg-3	(*R*,*R*)	7.897	9.1768	8.518
	(*P*,*R*)	7.894	9.1772	8.517
	(*P*,*P*)	7.891	9.1765	8.516
aug-pcSseg-4	(*R*,*R*)	7.8988	9.1779	8.5204
	(*P*,*R*)	7.8984	9.1778	8.5206
	(*P*,*P*)	7.8981	9.1778	8.5208
DKS^75^	CHF	7.91	9.17	8.51

In summary, within the transparent region, the electric dipole polarizability α_*αβ*_(ω) of a molecule has sometimes been expressed in the form of integrals over three-dimensional space of second-rank density functions, e.g., (***r***, ω) in eq 10. The disadvantage of definitions employed so far is that they depend on the origin of the coordinate system, which makes them unsafe from the physical viewpoint and unsuitable to weigh the contribution of different molecular regions to α_*αβ*_(ω) components.

Much more promising appears the alternative proposal of a translationally invariant density function in  based on the current density tensor , eqs 17 and 20, associated with the current density vector field induced by time-derivative of the electric field of a monochromatic light beam. Its spatial integral yields the components of the α_*αβ*_^(*R*,*P*)^(ω) polarizability within mixed dipole length-dipole velocity gauge, coinciding with α_*αβ*_^(*R*,*R*)^(ω) customarily considered, if off-diagonal hypervirial theorems, eq 19, are satisfied, a condition virtually met in actual calculations by employing high-quality basis sets.

The theoretical methods investigated in our paper are invariant of the origin within the algebraic approximation (for any basis set), as proven via numerical tests, and quite practical for computing and rationalizing dynamic polarizabilities. They are also applicable in the limit ω → 0 to calculate static polarizabilities.

The computational scheme implemented in the present research has been applied to investigate the carbon monoxide and nitrogen molecules by using very large basis sets. The results obtained have been represented by means of diverging color maps documenting efficiency and power of electric dipole polarizability density plots defined via the  current density tensor. In particular, they explain why the perpendicular component α_⊥_(0) is smaller than α_∥_(0) in both molecules, and why the two components change with the radiation frequency ω.

Future applications of the theoretical procedure reported here are expected to provide important contributions to the comprehension of mechanisms which operate alongside polarization of the electronic cloud in atoms and molecules responding to static and optical electric fields, as well as fundamental information on the role played by different molecular regions. In particular, integration of the  density function in various domains of the electron cloud defined by ad hoc criteria, e.g., AIM Bader’s theory^36^ or the LoProp method,^22^ would be very useful for assessing local quotas of the total electric dipole polarizability of a molecule in quantitative terms.
